# Supplementary material for: Population-scale organization of cerebellar granule neuron signaling during a visuomotor behavior
Source: Sci Rep. 2017 Nov 24;7:16240. doi: 10.1038/s41598-017-15938-w (PMC5701187; doi:10.1038/s41598-017-15938-w)
Supplement: Supplementary file 1 — Supplementary Information [file 41598_2017_15938_MOESM1_ESM.pdf]

SUPPLEMENTARY INFORMATION

Title: Population-scale organization of cerebellar granule neuron signaling during a visuomotor behavior

Authors: Sherika J.G. Sylvester, Melanie M. Lee, Alexandro Ramirez, Sukbin Lim, Mark S. Goldman, Emre R. F. Aksay

SUPPLEMENTARY INFORMATION

Supplementary Figure 1: Spectral fingerprinting procedure for identifying the inner granule layer (IGL) after OGB dye loading.

(**a**) Before OGB loading, GFP-positive granule cells in the inner granule layer (IGL) are easily identified with 930 nm excitation in Tg(*gata1*:GFP) zebrafish. The shown image plane contains large portions of the right cerebellar lobe and parts of the left lobe (dashed: midline); granule cell axons are located rostrally and medially. The scale bar, here and below, is 10 µm.

(**b**) After OGB injection into the right lobe, the IGL has similar intensity to the rest of the cerebellum, both at 930 nm (left) and 790 nm (right) excitation. Note, however, the slightly elevated intensity in the IGL at 930 nm.

(**c**) A pixel-by-pixel difference (Methods) of the 930 nm and 790 nm images highlights the IGL.

(**d**; left) To identify the borders of the IGL, a histogram of pixel intensity differences (black) is compared to that expected from noise; the noise histogram is generated by mirroring negative difference values about zero (grey; gaussian fit shown in dashed orange). Those difference values a standard deviation or greater from zero were considered candidate GFP-positive pixels (green box). (right) Identified regions passing size and contiguity cutoffs (green, Methods) align closely with the outlines of the IGL identified in panel a (black).

(**e-g**) Outlines of the region identified as containing GFP-positive granule neurons (green) overlaid onto the images in panels a-c.

Supplementary Figure 2: Targeting granule neurons for antidromic activation.

To determine how changes in granule cell firing were coupled to changes in the somatic calcium concentration, we sought to identify a location for electrical stimulation of granule cell axons that would enable antidromic activation of granule neurons in the absence of direct depolarization to the soma or secondary input from mossy fibers or other cerebellar neuronal classes. Here we show that targeting of the caudal molecular layer in one half of the cerebellum allows selective access to many of the granule cells with somata in the other half of the cerebellum. To do so, we targeted caudal lobe commissure parallel fibers[1] for electroporation with 3 kD Texas Red Dextran (D-3329, Life Technologies; 10 mM in distilled water; 7 dpf *nacre*). Electroporation via a fine glass capillary (1 second long train of 2 msec 70 V pulses delivered at 200 Hz) resulted in immediate loading of cells and tissue near the poration site (“X”). Over a course of ten minutes, dye diffused in parallel fibers away from the poration site

to label granule cell somata located on either side of the midline within the *eminentia granularis* and lateral portions of the IGL. Some parallel fibers were also seen to exit the cerebellum on both sides (green arrows) and terminate in rhombomeres 1 and 2 (not shown). For the antidromic activation experiments found within the main text, depolarizing electrical pulses were therefore delivered at locations similar to the location of the electroporation shown here, thereby minimizing activation of a larger portion of the cerebellar circuit and ensuring that changes in somatic fluorescence intensity were directly driven by changes in granule cell firing.

Supplementary Figure 3: Fitting granule cell responses

(**a**) Fluorescence responses were modeled as encoding a low-pass filtered optokinetic velocity signal that then gets filtered through a calcium impulse response function that models the kinetics of calcium buffering. Under this model, the applied optokinetic velocity signals (blue) were convolved first with a low-pass (exponential decay) filter of time constant τ_p (maroon) and then with a second low-pass (exponential decay) filter of time constant τ_c representing the effects of intrinsic calcium buffering (orange). The time constant τ_c was empirically determined via glutamate stimulations (see CIRF time constant estimation section) and the time constant τ_p was fit to the fluorescence and applied optokinetic velocity traces.

(**b**) Histogram of model fit r^2 values for all cells. Only those with fit goodness values equal to on in excess of 0.5 were used in subsequent analyses.

(**c**) Histograms of residual autocorrelation to assess remaining signal. The generally lower values for cells with fit r^2 values less than 0.5 suggest there was relatively little signal on those cells not captured by the model.

Supplementary Figure 4: Optokinetic responses of neurons in the vestibular complex

(**a**) Representative cycle-triggered fluorescence responses (green) of four neurons in the vestibular complex of the larval zebrafish during optokinetic stimulation (blue). Gray bars denote the time of change of stimulus velocity. Model fits are in orange.

(**b**) Histogram of the number of neurons within each response type. Grey dashed lines at rectification index (RCI) values of 2/3 and 4/3 denote response type boundaries. Green indicates positive and red indicates negative rectification for half- and full-wave responses; for reciprocal cells, the two colors indicate whether the positive (green) or negative (red) epoch achieved the greatest magnitude. (Top) Model fits to typical neurons in each subgroup with ipsiversive

movement sensitivity (i.e. excitatory response to ipsiversive movement and/or inhibitory response to contraversive movement); those with contraversive movement sensitivity are also included in the histogram. (**c**) Polar plot indicating the relative contribution of the fit coefficients A_{ipsi} and A_{contra}. Grey dashed lines denote the RCI response group cutoffs in polar coordinates.

Supplementary Figure 5: Spatial patterning in pairwise correlations

The spatial dependence of the strength of functional association between responsive granule cells was determined by calculating for every pair the correlation (or anticorrelation) between activity time series and pairwise distance (blue). For comparison, we performed the same measurement for granule cells that were not responsive during the optokinetic behavior (black). Data were grouped into 20 bins with equal sample numbers, and then the mean +/- s.e.m. index difference was plotted against the mean separation distance for each bin. Statistics were calculated after binning.

Supplementary Table 1: Summary of statistics for spatial patterning in functional metrics

Summary statistics for pairwise analyses of spatial patterning in the rectification index (RCI), signed rectification index (RSI), and direction sensitivity index (DSI) along the rostro-caudal axis (RC), medio-lateral axis (ML), and major and minor axes of the granule population. Bootstrap and robust regression analysis revealed that all significant trends were robust to outliers except the trend for RSI along the ML axis.

Supplementary Table 2: Comparison of rectified classification to that of classification system of Duensing and Schaefer

In this work we have unified the various responses observed in the granule layer under the framework of rectified signaling. Here, for completeness, we tabulate how this organizational system compares to that of Duensing and Schaefer[2-5], which was developed as a way to group the responses observed in vestibular pathways following bilateral or unilateral labyrinth stimulation. For simplicity, we only relate the reciprocal and half-wave rectified classes to descriptions where eye and head movement sensitivities are in opposing directions. *Abbreviations:* MC, minimal change; E_I/E_{II}, ipsiversive/contraversive eye movement sensitivity; H_I/H_{II}, ipsiversive/contraversive head movement sensitivity.

Supplementary References

1 Volkmann, K., Rieger, S., Babaryka, A. & Koster, R. W. The zebrafish cerebellar rhombic lip is spatially patterned in producing granule cell populations of different functional compartments. *Dev Biol* 313, 167-180, doi:S0012-1606(07)01438-8 [pii] 10.1016/j.ydbio.2007.10.024 (2008).

2 Duensing, F. & Schaefer, K. P. [The activity of various neurons of the formatio reticularis of the unfettered rabbit during head turning and vestibular stimulation]. *Archiv fur Psychiatrie und Nervenkrankheiten, vereinigt mit Zeitschrift fur die gesamte Neurologie und Psychiatrie* 201, 97-122 (1960).

3 Gernandt, B. Response of mammalian vestibular neurons to horizontal rotation and caloric stimulation. *Journal of neurophysiology* 12, 173-184 (1949).

4 Shimazu, H. & Precht, W. Tonic and kinetic responses of cat's vestibular neurons to horizontal angular acceleration. *Journal of neurophysiology* 28, 991-1013 (1965).

5 Ryu, J. H., McCabe, B. F. & Funasaka, S. Types of neuronal activity in the medial vestibular nucleus. *Acta oto-laryngologica* 68, 137-141 (1969).

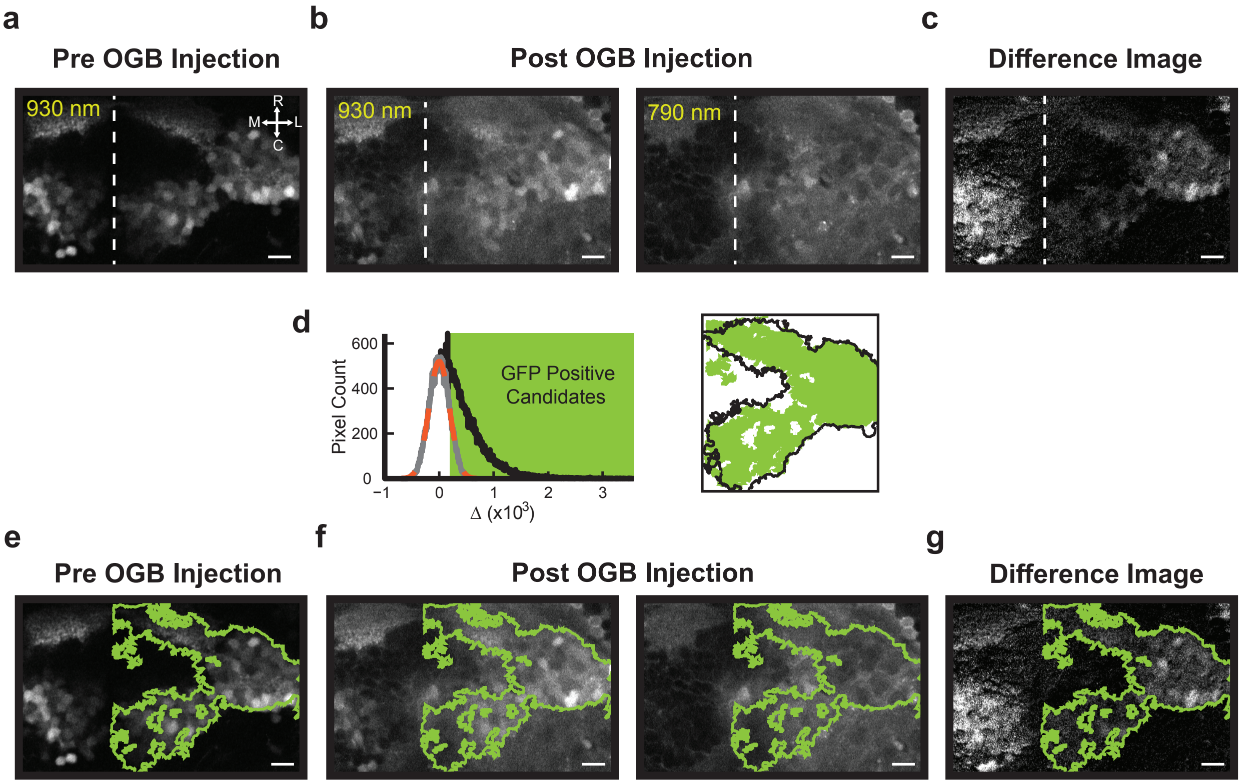

Supplementary Figure 1

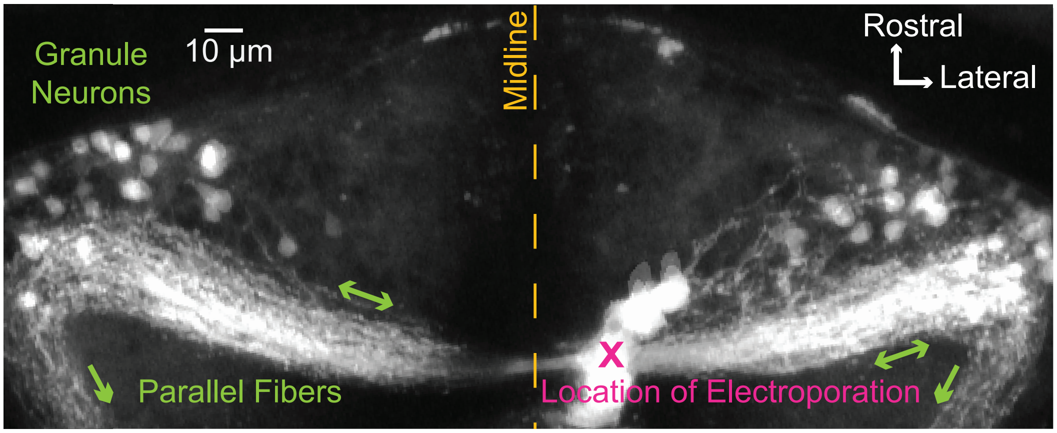

Supplementary Figure 2

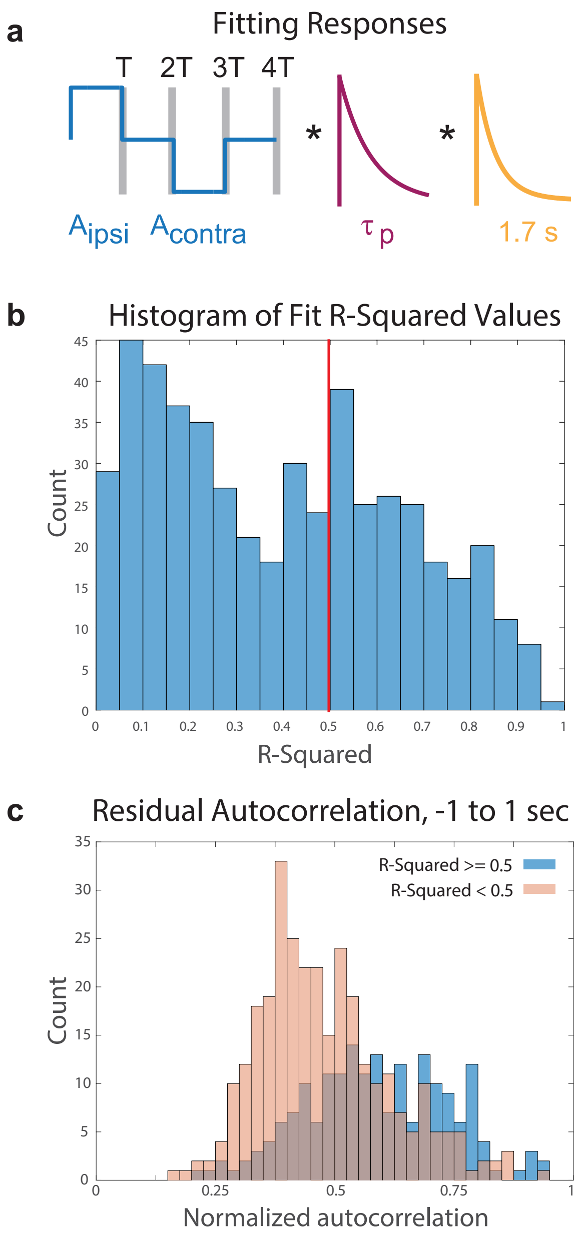

Supplementary Figure 3

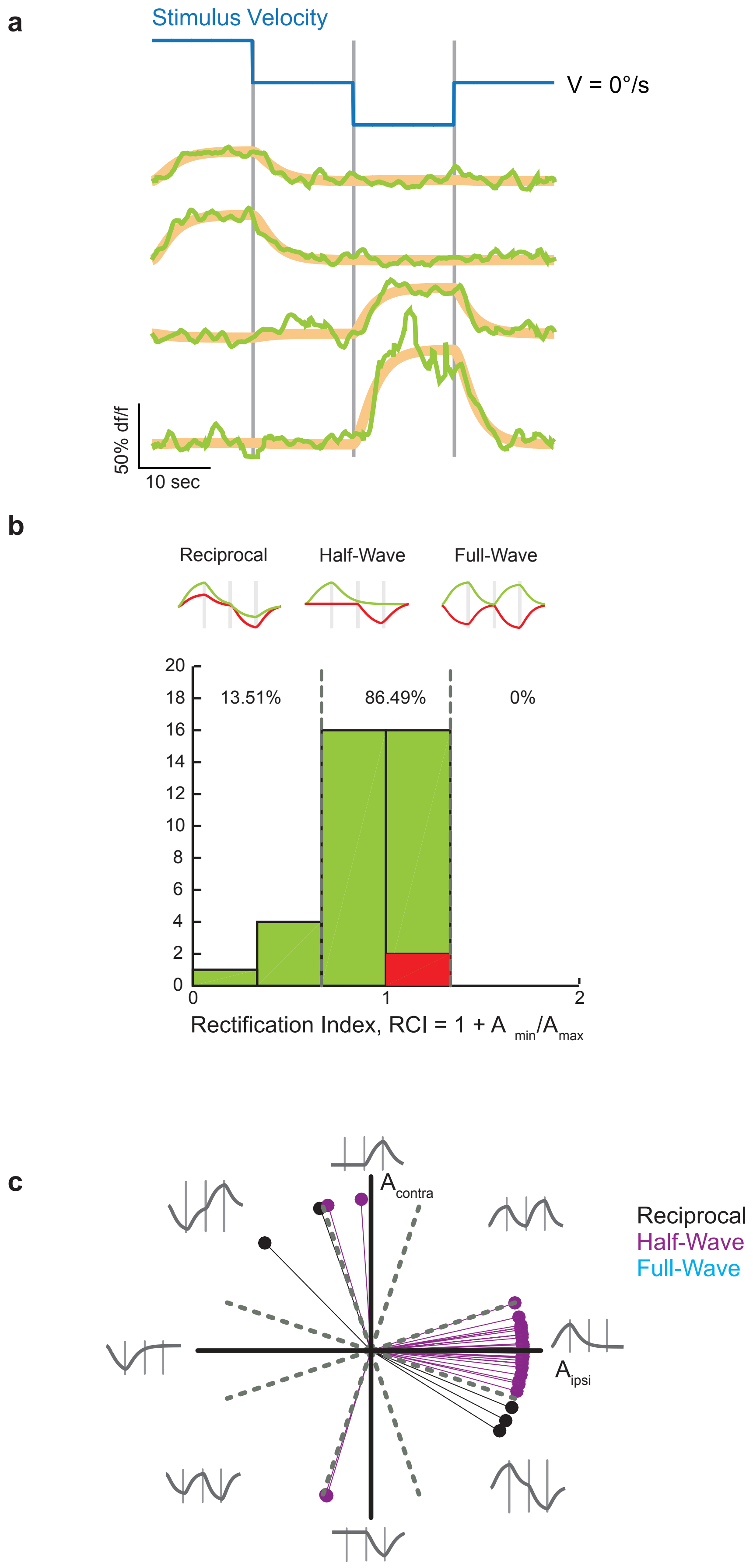
a
Stimulus Velocity
V = 0°/s
50% df/f
10 sec
b
Reciprocal
Half-Wave
Full-Wave
13.51%
86.49%
0%
Rectification Index, RCI = 1 + A min/Amax
c
Acontra
Aipsi
Reciprocal
Half-Wave
Full-Wave

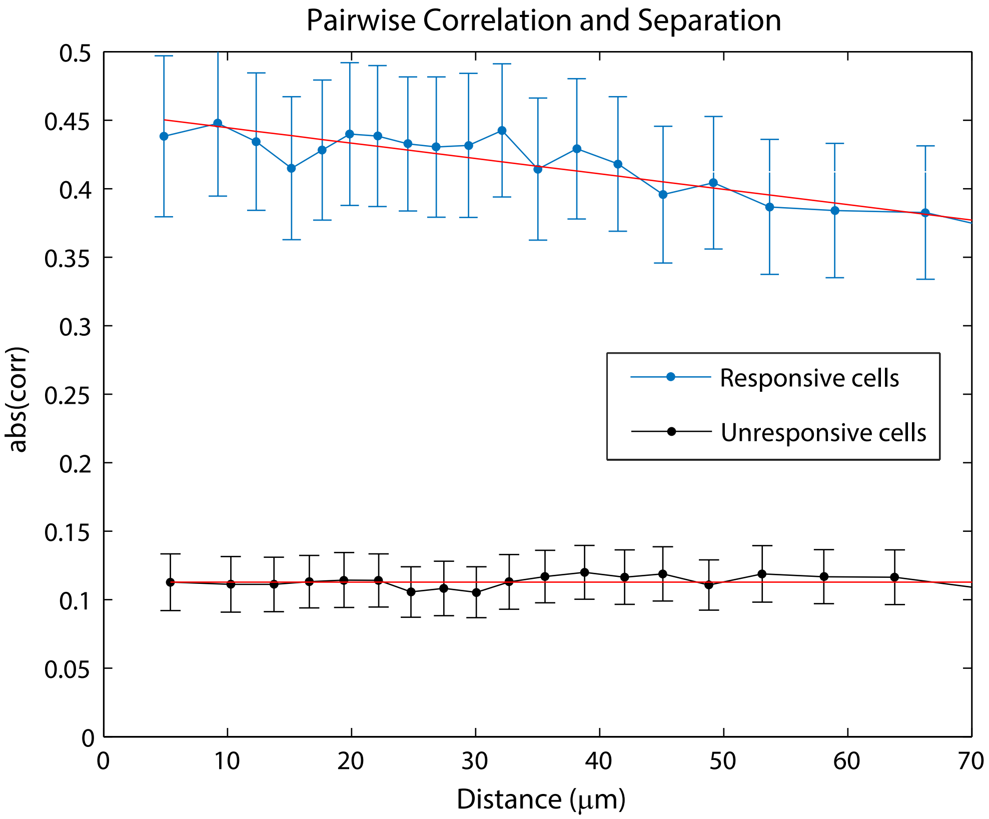

Supplementary Figure 4

		RCI	RSI	DSI
RC axis	Slope	-8.10E-04	-2.51E-03	-2.34E-03
	SRC	1.65E-02	-1.33E-02	-3.95E-02
	P value	4.30E-01	5.25E-01	5.90E-02
ML axis	Slope	-3.23E-03	-3.36E-03	1.31E-03
	SRC	-8.41E-02	-1.71E-01	3.01E-02
	P value	5.64E-05	1.10E-02	1.51E-01
Major Axis	Slope	1.80E-03	4.88E-03	6.69E-04
	SRC	4.17E-02	6.46E-02	-1.05E-02
	P value	4.64E-02	1.99E-03	6.15E-01
Minor Axis	Slope	-7.64E-03	-1.86E-02	-1.38E-03
	SRC	-7.52E-02	-1.18E-01	1.40E-02
	P value	3.22E-04	1.41E-08	5.03E-01

Supplemental Table 1

**Rectification Class**	**Ipsilateral**	**Contralateral**	**Type**	**Subtype**
Ipsiversive Reciprocal	Increase	Decrease	$E_I H_{II}$	H_{IIA}
Ipsiversive Positive Half-Wave	Increase	MC		H_{IIB}
Ipsiversive Negative Half-Wave	MC	Decrease		H_{IIC}
Contraversive Reciprocal	Decrease	Increase	$E_{II} H_I$	H_{IA}
Contraversive Positive Half-Wave	MC	Increase		H_{IB}
Contraversive Negative Half-Wave	Decrease	MC		H_{IC}
Positive Full-Wave	Increase	Increase	III	-
Negative Full-Wave	Decrease	Decrease	IV	-

Supplemental Table 2
